# Stigma and self-perceived burden in postoperative liver cancer patients: the mediating role of financial toxicity

**DOI:** 10.3389/fpsyt.2025.1481752

**Published:** 2025-02-26

**Authors:** Yue Zhang, Yiqin Cao, Yanyan Wang, Di Wang, Hongfang Ye, Chunlei Nie

**Affiliations:** ^1^ Department of Nursing, Nanjing Drum Tower Hospital, Affiliated Hospital of Medical School, Nanjing University, Nanjing, China; ^2^ School of Nursing, Nanjing University of Chinese Medicine, Nanjing, China; ^3^ Department of Vascular Surgery, Nanjing Drum Tower Hospital, Affiliated Hospital of Medical School, Nanjing University, Nanjing, China; ^4^ School of Humanities, Southeast University, Nanjing, China

**Keywords:** liver cancer, financial toxicity, stigma, self-perceived burden, mediating effects

## Abstract

**Purpose:**

To investigate the mediating effect of financial toxicity on stigma and self-perceived burden in patients with liver cancer after surgery.

**Methods:**

Using a convenience sampling method, 236 postoperative liver cancer patients treated at a tertiary hospital in Nanjing from April 2024 to July 2024 were selected for the study. Questionnaires were administered, including a general information survey, the Social Impact Scale (SIS), the Self-Perceived Burden Scale for Cancer Patients (SPBS-CP), and the Comprehensive Score for Financial Toxicity-Functional Assessment of Chronic Illness Therapy Version 2 (COST-FACIT-V2). Data were analyzed using SPSS 22.0 for descriptive statistics, correlation analysis, and regression analysis. MPlus 8.3 was employed to examine the mediating effect of financial toxicity between stigma and self-perceived burden, and the Bootstrap method was used to test the significance of the mediation effect.

**Results:**

The self-perceived burden score, stigma score, and financial toxicity score were (31.72 ± 7.52), (58.92 ± 8.69), and (18.62 ± 6.80), respectively. The financial toxicity and self-perceived burden were negatively correlated (r=−0.270,P=0.001). There was a positive correlation between stigma and self-perceived burden (r=0.586,P=0.000). Financial toxicity partially mediated the relationship between stigma and self-perceived burden, accounting for 4.84% of the total effect.

**Conclusion:**

Stigma can influence the self-perceived burden of liver cancer patients through financial toxicity. Clinical nurses should prioritize alleviating patients’ stigma while also paying close attention to their financial toxicity status, providing feasible assistance in a timely manner to reduce their self-perceived burden.

## 1 Introduction

Primary liver cancer has become a global health challenge, ranking sixth among the common cancer types and third among the causes of cancer deaths globally. It is characterized by insidious onset, high malignancy, and high mortality (1). According to statistics, in 2020, the number of new liver cancer cases worldwide is approximately 906,000, and the number of new liver cancer deaths is 830,000, whereas China accounts for approximately half of the world’s primary liver cancer patients (2). Hepatocellular carcinoma (HCC) is the most prevalent form of liver cancer, accounting for approximately 75%–85% of cases, and the main risk factor for liver cancer in the Chinese region, in comparison with the main cause of liver cancer in Western patients, which originates from alcohol-associated liver cirrhosis, are Hepatitis B virus or hepatitis C virus infection secondary to cirrhosis (3). According to a survey, 69.9% of HCC patients in China have a background of HBV infection, 5.2% have a history of hepatitis C virus infection, and 5.8% have a history of both (4). Surgery is one of the most important treatment methods for liver cancer. Despite the great development of medical technology in recent years, the long-term survival rate of patients with liver cancer has been effectively improved (5), but after surgery, patients still have to bear the physical adverse reactions brought by the disease, but also bear the inconvenience of follow-up regular treatment, and the substantial psychological pressure resulting from the increased financial burden on their families. Moreover, they are susceptible to self-perceived burden.

Self-perceived burden (SPB) refers to the psychological reaction of patients who feel that they are a burden to others because of the impact of their illness and care needs on others (6). It is a well-documented phenomenon that patients facing serious diseases often experience SPB (7). Influenced by family concept and culture, when family members face life-threatening diseases, the family tends to unite in combatting the crisis, and patients may perceive themselves as a burden to their family members. The energy and economic effort expended by caregivers can engender a sense in patients that they are dragging down their family members, resulting in varying degrees of psychological distress, such as anxiety, depression, and loss of dignity (8). Approximately 70% of hepatocellular carcinoma patients in China carry hepatitis B (4). Due to the lack of knowledge of viral hepatitis in the current public health policy, the society often harbors a kind of avoidance and fear of patients with hepatitis B. Although a large proportion of patients can still be reintegrated into the workplace as when they are healthy after surgery, the return of hepatoma patients to the workplace, especially those with hepatitis B, may be subjected to heavy resistance in normal production work and social life due to discrimination (9). This phenomenon is not merely attributable to employers’ preconceived notions; rather, it stems from a deep-rooted societal reluctance to embrace individuals with hepatitis B, which, as previously mentioned, is not without foundation, coupled with the fact that liver cancer patients are easily troubled by symptoms and fear of recurrence, which makes it easy for liver cancer patients to feel stigma (10). Stigma is referred to as “a heavily stigmatized attribute” that primarily involves psychological and emotional stress, which can hinder treatment and negatively impact patients’ quality of life (11). When patients develop a sense of shame, their life satisfaction and overall well-being decrease, and they also experience a decline in mental health and adverse coping behaviors will appear, which are detrimental to their physical and mental health (6). The study by FAN et al. (12) found that patients’ sense of stigma is highly correlated with their SPB; due to self-stigmatization, patients feel burdened by others and have negative feelings of uselessness and thus fall into a deeper sense of shame, thus falling into a deeper vicious circle. Li et al.’s study also demonstrated that patients’ SPB has a significant impact on stigma; patients may fear being stigmatized by others due to significant physical changes, reduced mobility, and the financial burden of treatment. In addition, the patient’s high dependence on the caregiver due to changes in the condition may lead to feelings of inferiority and guilt, which undoubtedly further aggravate the patient’s social isolation and negative emotions in interpersonal interactions and ultimately exacerbate the sense of stigma (13).

Financial toxicity (FT) has been validated as an important risk factor for SPB in cancer patients (14). FT refers to the negative impact on patients and their families caused by the medical costs of treating disease in cancer patients, including objective financial burden and subjective economic hardship (15). Liu et al.’s (16) study found that lung cancer patients often face FT challenges due to treatment, which may further make patients feel that they are a burden to their families, thus deepening their burden and stigma. As patients with high SPB have more significant perceived FT, which in turn increases their subjective psychological pressure, patients may fall into a vicious circle of disease burden and negative emotions, which greatly harms the prognosis of patients (17). Concurrently, FT has been identified as a contributing factor to stigma. A study of breast cancer stigma in Japan found that economic factors significantly influenced patient stigma (18). A survey of factors influencing stigma in cancer patients in China supports that among patients with moderate to high levels of stigma, the factor with the highest average patient-reported score is “financial insecurity,” which is highly consistent with the concept of FT (19). Liver cancer patients and their primary caregivers inevitably work fewer hours during periodic visits, which can lead to reduced income (20). The financial burden of cancer treatment, coupled with the psychological distress of the fear of relapse, can result in patients and their families becoming entrapped in a state of financial toxicity, characterized by the depletion of their savings and the subsequent need to incur debt to continue treatment. In extreme cases, some patients may even opt to discontinue their treatment or even resort to self-harm (21). However, there are insufficient studies on groups with high stigma and high SPB, such as patients with liver cancer.

Taken together, the stigma, SPB, and FT of postoperative hepatoma patients have clinical values that cannot be ignored. The current literature mainly discusses the correlation between patients’ stigma, SPB, and FT (22), and analyses of the influencing mechanisms among the three are relatively lacking. Therefore, this study hypothesizes that there is a correlation between patients’ stigma and SPB after liver cancer surgery, and FT can adjust the influence of stigma level on SPB, so as to further explore the mediating role of FT in the relationship between patients’ stigma and SPB and to provide theoretical reference for the development of targeted interventions for patients with liver cancer who are suffering from both disease and psychological pain. We will further explore the mediating role between FT and SPB in order to provide a theoretical reference for the development of targeted interventions for liver cancer patients who are suffering from disease and psychological pain.

## 2 Materials and methods

### 2.1 Sampling and participants

The sample size of this study was calculated according to GORSUCH’s sample size calculation method (23), which is 5–10 times the size of the dependent variable, taking into account a 10% inefficiency rate. The calculated sample size is 149–298 cases, which meets the requirements of the sample size needed to construct the structural equation model. The study was conducted in a large Grade III hospital with sufficient sample size in Nanjing; a total of 236 patients with liver cancer who were admitted to a Grade III hospital in Nanjing from April 2024 to July 2024 were selected using the convenience sampling method as the study objects. A total of 250 questionnaires were sent out, and 236 were valid, with an effective recovery rate of 94.4%.

Inclusion criteria: (1) postoperative patients diagnosed with primary liver cancer by pathological tissue with reference to the relevant diagnostic criteria of the Diagnostic and Treatment Criteria for Primary Liver Cancer (2019 edition) (24); (2) age ≥18 years old; (3) the patients were aware of their condition;(4) be able to complete the questionnaire in written or oral form;(5) informed consent and voluntary participation.

Exclusion criteria: (1) combination of primary tumors elsewhere or severe organ dysfunction of the heart, brain, or kidneys; (2) complicated with severe postoperative complications of liver cancer; (3) combined with serious mental disorders, cognitive disorders, language disorders, and audio-visual impairments.

### 2.2 Research instruments

#### 2.2.1 Sociodemographic information

The sociodemographic information collected in this study included gender, age, education level, marital status, number of children, payment method of medical expenses, years of illness, chronic medical history, personal average monthly income, and treatment method.

#### 2.2.2 Social impact scale

This scale was developed by Fife et al. (25) in 2000 and can be used to measure the stigma of cancer patients. Pan et al. (26) translated it into Chinese in 2007, with 24 items in four dimensions, namely, economic discrimination, social isolation, internal shame, and social exclusion. It uses Likert’s four-point scoring method, in which “strongly agree” means 1 score, “strongly agree” means 4 score, and the higher the sum of scores of all dimensions, the stronger the stigma of patients. The total Cronbach’s α coefficient of the scale was 0.85~0.90.

#### 2.2.3 Self-perceived burden assessment scale for cancer patients

Ren et al. (27) translated the scale into Chinese and developed it more targeted at Chinese cancer patients, including 21 items in four dimensions, namely, care burden, economic burden, psychological and emotional burden, and treatment burden. According to Likert’s fifth-point scoring method, the higher the score, the more serious the SPB, and the score <30 indicates no obvious burden. 30~<50 was classified as mild burden, 50~<70 as moderate burden, and ≥70 as severe burden. Cronbach’s α coefficient of this scale was 0.938.

#### 2.2.4 Comprehensive score for financial toxicity-functional assessment of chronic illness therapy version 2

Using the Chinese version scale of Chan et al. (28), there are a total of 12 items in the four dimensions of financial items, resource items, impact items, and overall FT. The 11 items in the first three dimensions are scored, and the Likert’s fifth-point scoring method is adopted, from 0 to 4 points, indicating “not at all” to “very much”. From 0 to 4 points in order of “not at all” to “very much”, the total score of 0 to 44 points, the lower the score, the higher the FT. A total score greater than 25 was classified as no FT, 14–25 as mild FT, 1–13 as moderate, and 0 as severe FT. The Cronbach’s α for this scale was 0.875.

### 2.3 Investigation method

Prior to the study, the researchers received uniform training to ensure that all researchers had the same understanding of all items on the scale. Before starting the survey, the researcher explained the purpose of the study, explaining that the study was based on patients’ volition and that patients were allowed to voluntarily withdraw at any stage of the study. Completion of the questionnaire was started independently after the patients signed the informed consent form, and the dyslexic patients completed the questionnaire after a uniform language explanation. Once the questionnaires were completed, the research team checked the questionnaires, and in case of omission or missing items, the subjects were asked to recomplete the questionnaires and check them to exclude the questionnaires with obvious errors in the data.

### 2.4 Statistical analysis

Data were entered by one researcher using EXCEL and checked again by another researcher. Data were analyzed using SPSS 22.0 and MPlus 8.3 statistical software. The entered measures were described by mean ± standard deviation if they conformed to normal distribution, whereas those that do not conform to normal distribution are described by median and quartile; the included counts were described by frequency counts and constitutive ratios; and Spearman’s analysis was used to test the correlation between FT, stigma, and self-perceived burden of illness. Mplus8.3 was used to construct the mediation model, the Bootstrap method was used to verify the mediation effect, test level p < 0.05 indicated that the difference was statistically significant.

## 3 Results

### 3.1 Sociodemographic variables

A total of 236 patients with liver cancer after surgery were collected in this study, and the general data results with statistical differences were shown in **Table 1**.

**Table 1:** Sociodemographic variables (n=236).

Item	Variables	n (%)	M (P25, P75)/x ± s	P	Item	variables	n (%)	M (P25,P75)/x ± s	P
Education	Primary and below	76 (32.20)	33 (26,39)	**	Working state	On the job	59 (25.00)	39 (24,33)	**
	junior high school	81 (34.32)	33 (25,39)			Unemployment	94 (39.83)	33 (26,40)	
	Vocational secondary/high school	49 (20.76)	29 (23,34)			Retirement	83 (35.17)	32 (25,37)	
	College degree and above	30 (12.72)	26 (24,35)		Average monthly earnings (RMB)	1,000 and below	40 (16.95)	33 (28,40)	**
Location	Country	138 (58.47)	33 (26,40)	***		1,000-3,000	76 (32.20)	33 (25,38)	
	City	98 (41.53)	28.5 (25,34)			3,000-5,000	56 (23.73)	32.5 (25,37)	
Payment	Self-financed	34 (14.40)	40 (35,46)	***		5,000 and above	64 (27.12)	27 (24,32)	
	Resident medical insurance	140 (59.32)	30 (25,35)		Stage of disease	I/II	110 (46.61)	30 (25,37)	**
	Employee medical insurance	62 (26.27)	30 (25, 36)			III	89 (37.71)	31 (25,35)	
Chronic disease	Yes	218 (92.37)	32 (25,38)			IV	37 (15.68)	36 (25,42)	
	No	18 (7.63)	25 (22,26)		Clinical trials	Yes	24 (10.17)	25 (22,28)	***
Therapy method	1	185 (77.97)	43 (30,48)	***		No	212 (89.83)	32 (25,38)	
	2	46 (20.34)	36 (32,41)		Targeted drug	Yes	32 (13.56)	38.5 (25.75,43)	**
	3 and above	5 (1.69)	30 (25,35)			No	204 (86.44)	31 (25,36)	

**means p<0.05; ***Means p < 0.001.

### 3.2 Correlation analysis of the studied variables

In this study, patients with liver cancer had a score of (58.92 ± 8.69) for stigma, (31.72 ± 7.52) for SPB, and (18.62 ± 6.80) for FT. The scores of various dimensions of the scale are shown in **Table 2**. The results of Spearman’s correlation analysis showed that patients had a positive correlation between the score for stigma and the score for SPB (r=0.638, P= 0.000), the stigma score was negatively correlated with FT score (r=–0.168, P=0.01), and the FT score was negatively correlated with the SPB score (r=−0.309, P=0.000).

**Table 2:** Stigma, self-perceived burden, and financial toxicity scores of patients with liver cancer after surgery (n=236,x ± s).

	Total score	Sub-item average score
**Stigma**	58.92 ± 8.69	2.46 ± 0.36
Economic discrimination	22.23 ± 6.93	2.47 ± 0.77
Social isolation	7.86 ± 2.49	2.62 ± 0.83
Inner shame	12.25 ± 3.80	2.45 ± 0.76
Social exclusion	16.59 ± 6.51	2.37 ± 0.93
**Self-perceived burden**	31.72 ± 7.52	1.51 ± 0.36
Care burden	9.66 ± 3.29	1.38 ± 0.47
Economic burden	7.16 ± 2.84	1.79 ± 0.71
Psychological and emotional burden	7.68 ± 1.68	1.28 ± 0.28
Treatment burden	7.16 ± 3.84	1.79 ± 0.96
**Financial toxicity**	18.62 ± 6.80	1.69 ± 0.62
Economic influence	2.48 ± 1.13	2.48 ± 1.13
Resource toxicity	3.72 ± 1.88	1.86 ± 0.94
Toxicity impact	12.40 ± 6.40	1.55 ± 0.80

Stigam, Self-perceived burden and financial toxicity are observation indicators, the non-bold parts are sub-dimensions.

### 3.3 Mediating effect of financial toxicity between stigma and self-perceived burden

**Figure 1:**
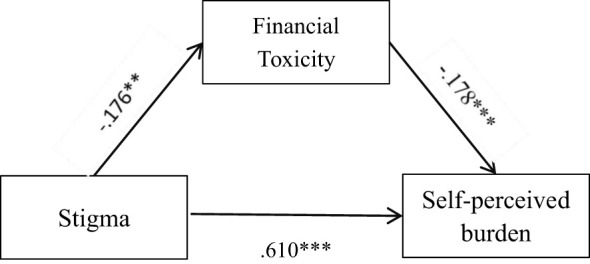
Mediating effect of financial toxicity on stigma and self-perceived burden in patients with liver cancer after surgery. The numbers represent the regression coefficients of the two variables connected by arrow lines. **means p<0.05; ***means p < 0.001.

The mediation effect test was performed using Mplus 8.3 with stigma as the independent variable, SPB as the dependent variable, and FT as the mediator variable. The results showed that FT had a significant mediating effect between the stigma and SPB, and the mediation model is shown in **Figure 1**. The 95% confidence interval (95% CI) of the mediating effect was calculated using the Bootstrap method with a sampling number of 5,000 to test the significance of the mediating effect, and the 95% CI was (0.007,0.074), which indicated that the mediating effect was significant. The values of the mediating effect are shown in **Table 3**. The mediated model showed that stigma had a direct positive predictive effect on SPB (β=0.610, P=0.000), FT had a direct negative predictive effect on SPB (β=−178, P=0.001), and stigma had a direct negative predictive effect on FT (β=−0.176,P=0.007). In addition, FT played a partial mediating role in the relationship between stigma and SPB in patients with liver cancer. Its mediating effect was (−0.176×−0.178) =0.031, and the total mediating effect was 0.031 + 0.610 = 0.641, accounting for 4.84% of the total effect. The mutual effects of variables are shown in **Table 4**.

**Table 3:** Mediating analysis of financial toxicity between stigma and self-perceived burden in patients with liver cancer after surgery (standardized).

Mediating effects	Value	BootSE	95%CI	Effect ratio (%)
Total effect	0.641	0.058		
Direct effect	0.610	0.058	[0.477, 0.704]	95.16
Indirect effect	0.031	0.017	[0.007, 0.074]	4.84

**Table 4:** Mediating effect of financial toxicity between stigma and self-perceived burden.

		Model fit indices			Parameter significance estimation			
Criterion variable	Predictor variable	R	R^2^	F	β	SE	t	P
Financial toxicity	Stigma	0.176	0.031	7.457	−0.176	0.05	−2.731	0.007
Self-perceived burden	Stigma	0.664	0.441	92.027	0.61	0.61	12.258	P<0.001
	Financial toxicity				−0.178	0.055	−3.57	P<0.001

## 4 Discussion

### 4.1 Status analysis of stigma, self-perceived burden, and financial toxicity in patients with hepatoma after surgery

According to the results of this study, the average SPB score of patients after liver cancer surgery was (31.72 ± 7.52), indicating that patients in this cohort exhibited mild SPB. The reasons were analyzed as follows: The analysis revealed that the majority of liver cancer diagnoses are made at a late stage, with approximately 15% of patients experiencing extrahepatic metastasis. Furthermore, novel surgical interventions and anti-tumor therapies have not yielded substantial improvements in 5-year survival rates, while concomitantly introducing various adverse effects to patients. Patients are concerned that the prognosis of the disease will not be as good as expected even after the “whole-family effort” treatment (29, 30). Secondly, periodic chemoradiotherapy makes it impossible for some patients to maintain their original stable job and may need to borrow money to pay for medical expenses. Families with low risk resistance may not be able to maintain their original ecology due to economic difficulties (31, 32); family’s financial distress due to the disease leads to the patient’s apologetic mentality and of self-doubt, resulting in a burden of self-feeling (33, 34). This reveals that healthcare professionals need to pay attention to the SPB of patients with malignant tumors, especially those with a single or no financial source. In their daily work, healthcare professionals must understand the patients’ family life background, discover abnormal psychological conditions in time, give positive guidance, and guide caregivers to accompany patients’ daily life, so as to increase the level of social support for the patients, reduce their SPB, and improve their physical and mental health and quality of life.

The patients in this study had a stigma score of (58.92 ± 8.69), which was at the level of mild stigma, slightly lower than the study by Wang et al. (10). Analysis of the reasons, 55.6% of male and 46.5% of female liver cancer deaths in China are caused by hepatitis B virus infection, patients with viral hepatitis are often discriminated against in the current society, and those who want to integrate into society may encounter significant barriers to employment opportunities and marriage due to hepatitis stigma and discrimination (35) It is therefore logical that there should be measures to reduce the level of stigma among patients, which is increasingly being recognized. Hepatitis B, as a public health problem, emotional support interventions by healthcare professionals are particularly important: we should correct patients’ treatment mentality, convey more knowledge about the disease to the patients, and help the patients to correctly recognize hepatitis B, and meanwhile create online patient communication channels, so that the patients can send a strong sense of loneliness and helplessness, which can reduce patients’ sense of disease shame, gain the patients’ sense of trust, and improve the satisfaction; at the same time, it will give the patients psychological support intervention, give the patients targeted encouragement, enhance the confidence of overcoming the disease, and greatly improve the clinical satisfaction.

In this study, the score of FT of patients after liver cancer surgery was (18.62 ± 6.80), suggesting the existence of mild FT in patients, which is slightly lower than that of domestic scholar Yuan et al.’s research on breast cancer patients (36). The reason is that most of the subjects in this study are hepatocellular carcinoma patients, Tu et al. (37) pointed out that the recurrence probability of patients with hepatocellular carcinoma within 2 years after surgery is 30%–50%, which means that patients still need to do adjuvant therapy and regular physical examination for a long time after discharge. In the meantime, the utilization of various diagnostic imaging procedures within the hospital setting, in conjunction with prescribed medications and scheduled follow-up appointments, has the potential to disrupt patients’ daily lives. This disruption, in turn, can lead to an escalation in medical expenditures and a heightened sense of trepidation regarding the potential for recurrence. At the same time, postoperative patients need to go to the hospital regularly for treatment, which may lead to a reduction in income and loss of stable jobs. Patients who are unable to take care of themselves or are partially unable to take care of themselves need a caregiver at their side, which may result in a reduction of the caregiver’s income. In turn, it can lead to greater financial hardship for the entire family and a more significant decline in the patient’s quality of life, both physically and psychologically. Disease is the most prominent poverty-causing factor among the poor people in this country. When individuals from economically disadvantaged backgrounds contract serious illnesses and their families lack the financial resources to cover the substantial out-of-pocket medical expenses, they frequently face a dilemma: either seeking treatment and incurring debt, or forgoing medical care. This predicament often traps them in a cycle of “disease-poor-disease”. Therefore, it is necessary to raise the health awareness of cancer prevention and treatment among the FT population, so as to achieve “early screening, early diagnosis, and early treatment” to save medical expenses as much as possible. The effective identification of individuals at high risk of FT, early prevention and risk management, improvement of the social security pocket function, and the establishment of a long-term mechanism for medical insurance for people who have escaped poverty are practical means of reducing the FT experienced by people in need.

### 4.2 There was a positive correlation between stigma and self-perceived burden in patients with liver cancer

In the present study, patients with greater stigma had stronger SPB, which is consistent with the findings of Gao et al. (38). Some patients have lifelong carrier and infectious viral hepatitis, are vulnerable to stigma in society, and suffer greater psychological pain and disease burden while undergoing the physical changes and therapeutic burdens brought about by liver cancer treatment itself (39). Meanwhile, the proportion of young men in the group is the highest (40), and this group is in the most difficult stage of carrying the burden of family and social responsibility; as the “backbone” of the family, the inconvenience caused by liver cancer surgery and follow-up treatment will inevitably affect the normal production and life order of patients. Reduction of personal income, increase of financial burden, aggravation of symptomatic distress, and the change of physical appearance all cause the stigma of patients, which in turn leads to the patients SPB and even may further affect the prognosis of patients in a vicious circle (9). Therefore, it is imperative to promote a positive societal perspective regarding liver cancer patients and individuals with viral hepatitis. This is essential not only for ensuring equitable treatment for patients and their families but also for disseminating health education information to the broader population.

### 4.3 Financial toxicity has mediating effect between stigma and self-perceived burden in postoperative liver cancer patients

The existence of FT in patients with liver cancer has been an undoubted fact; 40% of the patients in this study believed that they were currently suffering from varying degrees of FT distress. In addition to the objective economic expenditure, high expenditure on cancer treatment increases the psychological burden of patients, which is consistent with the concept of FT, which emphasizes that the economic burden of cancer increases the subjective suffering of patients (41). Patients with a high financial burden will have a strong psychological burden, including guilt for the family, worry about future treatment, and fear of uncertain prognosis. A significant association has been identified between cancer-related financial stress and an elevated risk of adverse psychological outcomes, such as SPB (42). When patients reduce the frequency of medical visits or even refuse treatment due to stigma, extreme negative emotions and weak physical state can exacerbate the degree of FT, which in turn will lead to a decline in the patient’s material standard, more significant mental health problems, and SPB (43). On the other hand, if patients overcome the stigma, cope with the disease with a good mental state and positive attitude, and maintain a healthy physical state and social function, FT will be reduced, resulting in a lower perceived burden and a more satisfactory prognosis (44). Therefore, reducing patients’ SPB not only needs urgent attention but also requires multi-field and multidisciplinary exchanges and cooperation. We should provide social support to patients while insisting on follow-up and continuity of care after discharge. In addition, reducing the burden of FT and SPB of patients can also be considered from a macro perspective: through the support of government policies, the proper use of the health insurance system, and the construction of a sound prevention and control system, early identification, effective intervention, and precise assistance for high-risk groups will reduce stigma and social alienation, reduce the FT of cancer, minimize SPB, and improve the quality of life of patients.

## 5 Conclusion

This study demonstrates that improving stigma in patients with liver cancer through early screening and rational intervention is beneficial in reducing the high incidence of FT, and it is also beneficial in decreasing SPB, which is important in enhancing the overall prognosis of patients. However, this study has some limitations. Firstly, the sample collection in this study was conducted in a single hospital, and the use of the convenience sampling method may limit the results representativeness, which should continue to expand the sample size to reduce the bias that exists in the study population. Secondly, although our study tried to exclude confounding factors that could seriously affect the results by limiting the exclusion criteria, considering that there are other independent factors that may have an impact, future studies may be conducted to find other impact factors on the patients. Furthermore, this study endeavored to maintain the subjects in an undisturbed environment to the greatest extent possible and assured the patients that the data would be kept completely confidential except for use in the study, but it was unavoidable that the results of the study might deviate from the actual situation based on the large number of subjective scales and the possibility that patients might have a social desirability bias.

## Data Availability

The original contributions presented in the study are included in the article/Supplementary Material. Further inquiries can be directed to the corresponding author.
